# Correlated color temperature and light intensity: Complementary features in non-visual light field

**DOI:** 10.1371/journal.pone.0254171

**Published:** 2021-07-12

**Authors:** Raquel Arguelles-Prieto, Juan Antonio Madrid, Maria Angeles Rol, Maria Angeles Bonmati-Carrion

**Affiliations:** 1 Chronobiology Lab, Department of Physiology, College of Biology, University of Murcia, Mare Nostrum Campus, IUIE, IMIB-Arrixaca, Murcia, Spain; 2 Ciber Fragilidad y Envejecimiento Saludable (CIBERFES), Madrid, Spain; CNRS, University of Strasbourg, FRANCE

## Abstract

An appropriate exposure to the light-dark cycle, with high irradiances during the day and darkness during the night is essential to keep our physiology on time. However, considering the increasing exposure to artificial light at night and its potential harmful effects on health (i.e. chronodisruption and associated health conditions), it is essential to understand the non-visual effects of light in humans. Melatonin suppression is considered the gold standard for nocturnal light effects, and the activation of intrinsically photosensitive retinal ganglion cells (ipRGCs) through the assessment of pupillary light reflex (PLR) has been recently gaining attention. Also, some theoretical models for melatonin suppression and retinal photoreceptors activation have been proposed. Our aim in this study was to determine the influence of correlated color temperature (CCT) on melatonin suppression and PLR, considering two commercial light sources, as well as to explore the possible correlation between both processes. Also, the contribution of irradiance (associated to CCT) was explored through mathematical modelling on a wider range of light sources. For that, melatonin suppression and PLR were experimentally assessed on 16 healthy and young volunteers under two light conditions (warmer, CCT 3000 K; and cooler, CCT 5700 K, at ~5·10^18^ photons/cm^2^/sec). Our experimental results yielded greater post-stimulus constriction under the cooler (5700 K, 13.3 ± 1.9%) than under the warmer light (3000 K, 8.7 ± 1.2%) (p < 0.01), although no significant differences were found between both conditions in terms of melatonin suppression. Interestingly, we failed to demonstrate correlation between PLR and melatonin suppression. Although methodological limitations cannot be discarded, this could be due to the existence of different subpopulations of Type 1 ipRGCs differentially contributing to PLR and melatonin suppression, which opens the way for further research on ipRGCs projection in humans. The application of theoretical modelling suggested that CCT should not be considered separately from irradiance when designing nocturnal/diurnal illumination systems. Further experimental studies on wider ranges of CCTs and light intensities are needed to confirm these conclusions.

## Introduction

Since life appeared on Earth, living beings have been exposed to continuous and cyclic environmental fluctuations, such as light-dark cycle or seasonal variations in photoperiod. Therefore, the existence of endogenous mechanisms to coordinate the physiology with the cyclic changes of environment has been favored through evolution. In mammals, the circadian timing system (CS) is composed by a hierarchically organized network of structures, including a main pacemaker, located in the suprachiasmatic nuclei (SCN) of the hypothalamus [1] and peripheral oscillators, located in most tissues and organs. The CS also includes input pathways that allow pacemakers to get synchronized with the environment, being the light-dark cycle the main *zeitgeber* to the central pacemaker. The CS output pathways are responsible for coordinating different processes and functions at behavioral, physiological and molecular levels [2], including the sleep-wake cycle, rhythmic hormone secretion (e.g. melatonin), rest-activity pattern, etc.

The anatomical input pathway starts at a small population of retinal ganglion cells that are intrinsically photosensitive (ipRGCs) due to the presence of melanopsin, a photopigment especially sensitive to blue light between 460–480 nm [3, 4]. Apart from their intrinsic photosensitivity, these cells also receive inputs from rods and cones (extrinsic pathway) [5–8]. Interestingly, ipRGCs not only send information to the central pacemaker, but are also involved in pupillary light reflex (PLR) [9–13] through their projections on the olivary pretectal nucleus (OPN) [14, 15], as well as in other circadian-related processes [16]. Different subtypes of ipRGCs with different functional roles have been described in mice and humans (recently reviewed in [17]). In mice, six subtypes have been characterized [M1-M6]. M1 ipRGCs (probably the mouse orthologous of human type 1) are involved in the SCN and OPN innervation. However, two distinct subpopulations of M1 subtype have been molecularly characterized, namely Brn3b+ and Brn3b- [14]. Interestingly, although all M1 ipRGCs are morphologically and electrophysiologically similar, these two molecularly different subpopulations co-exist and innervate different brain regions: M1 Brn3b– for SCN and M1 Brn3b+ for OPN [17]. Among all output pathways, pineal melatonin secretion is the best characterized rhythm, with a peak during the night and lower levels during the day [18–21]. This hormone exerts a prominent chronobiotic function and, in humans, also plays an important role in sleep promotion [22]. Its secretion follows an endogenous rhythm regulated by noradrenergic stimulation of the SCN, with maximum levels during the night and low levels during the day [23], and it is also acutely suppressed by light, especially in the range of 460–480 nm [24, 25].

Although melatonin is considered the gold standard for circadian light effects [26–29], the fact that M1 ipRGCs project, not only to the SCN, but also to the OPN [16], make pupillometry an attractive technique to directly assess non-visual effects of light at input pathways levels. It is therefore of interest to explore the possible relationship between both PLR and melatonin suppression in order to try to infer in humans the possible existence of ipRGCs subpopulations, each differentially innervating OPN and SCN.

The generalization of electrical lighting about 150 years ago, together with lifestyle in modern societies, have involved a progressive decrease in bright light exposure during the day, and an increase in artificial light exposure during the night. This, in conjunction with nightlife, shift work and transmeridian flights, has given rise to a phenomenon known as chronodisruption (CD), which involves a misalignment among physiological internal rhythms and between internal and external times [30, 31]. CD has been associated with several health impairments, such as metabolic syndrome [2, 32], cardiovascular disease [33], psychiatric, cognitive and affective impairments [34], premature aging [35] and even higher incidence of some types of cancer [36, 37].

Therefore, it becomes essential to deepen our knowledge on the effects of light, especially during the night, considering the irradiances and spectra most commonly used for commercial purposes. The relationship between light correlated color temperature (CCT), light levels and subjective comfort has been deeply studied [38, 39]. Regarding objective biological parameters, melatonin suppression and photoreceptors sensitivities have been implemented in different theoretical models [40–42] that have been recently standardized by the International Commission on Illumination (CIE S 026/E:2018) [43]. Recently, PLR has been proposed as a new marker of circadian photoreception [9, 11, 44–47]. However, the link between PLR and melatonin suppression has only scarcely been explored [48].

In this sense, we have explored, in two commonly used commercial light sources, the contribution of CCT on both melatonin suppression and melanopic response, measured by PLR, and aimed to determine whether these two tests could be used as equivalent or complementary. For that purpose, in this study we analyze the effects on melatonin suppression and PLR of two commercial light sources (CCT 3000 K and 5700 K) at one fixed light irradiance. Previously published mathematical models have been also applied to these and to a wider range of commercial light sources and intensities.

## Materials and methods

### Participants

To perform these experiments, sixteen volunteers (9 females, 23.8 ± 3.12 yr old, mean ± SD) were recruited. This study was approved by the University of Murcia Ethics Committee (CBE 121/2018) and was in line with the standards set by the *Declaration of Helsinki*. All participants gave written informed consent prior to participation. Volunteers were not taking any medication that could influence the results of the study. Besides, none of them had suffered vision surgery. Two days before each experimental session, they were asked to avoid caffeinated drinks and to follow regular sleep habits during the three weeks of study, although the compliance was not verified. No chronotype exclusion criteria were applied. All melatonin samples were taken during November and pupil light reflex measurements in December.

### Lighting sources

**Fig 1:**
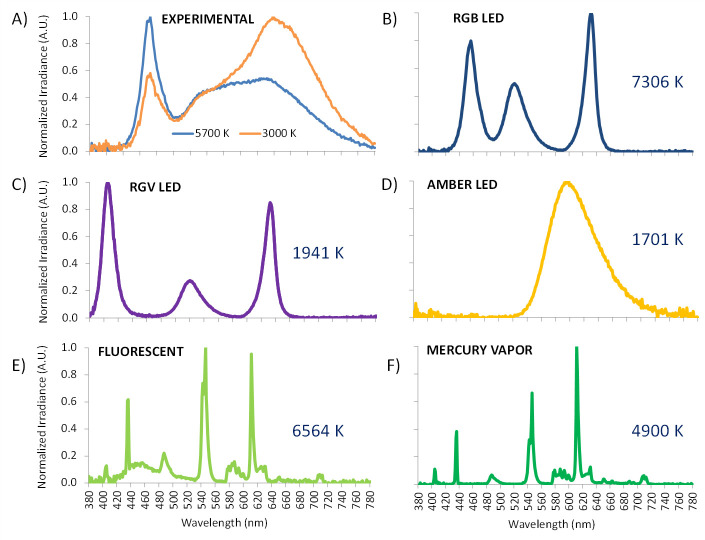
Spectral power distribution of the experimentally tested light sources: 5700 K (blue) and 3000 K (orange) (A) and mathematically assessed [42] light sources: red, green and blue (RGB) monochromatic LED light (7306 K) (B), the same prototype substituting blue for violet (RGV, 1941 K) (C), both of them manufactured at University of Murcia electronic facilities, and three commercial bulbs: amber LED (1701 K) (D), fluorescent light (6564 K) (E) and a mercury vapor bulb (4900 K) (F).

One smart LED panel (model squared Inspire 48W Gdansk, Leroy Merlin S.L., Lille, France) was employed. Correlated color temperature (CCT) was fixed in two conditions: at 3034 K (3000 K) and at 5669 K (5700 K), to investigate the effect of CCT on melatonin suppression and pupil light reflex. Spectra of both experimental lighting sources (Fig 1A) were measured with a visible-near infrared spectroradiometer model USB2000+ (Ocean Optics Inc., Florida, USA).

Light intensity was adjusted to remain as similar as possible among the two conditions in illuminance (569 vs 581 lux), irradiance (184 vs 181 μW/cm^2^) and photon flux (4.97·10^18^ vs 5.18·10^18^ photons/cm^2^/sec) for 5700 K and 3000 K, respectively.

### Melatonin suppression

**Fig 2:**
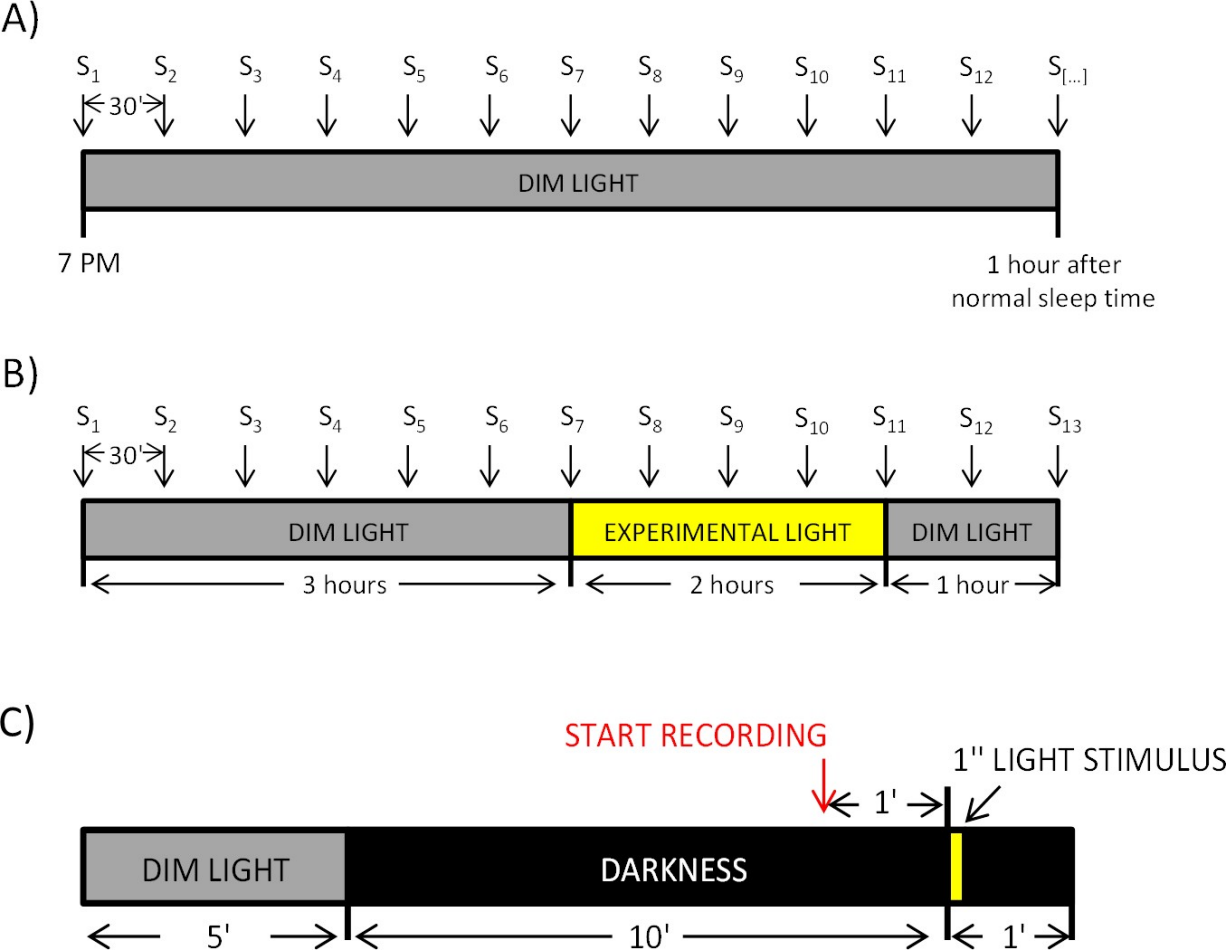
Melatonin suppression (A and B) and PLR (C) protocols. A) Dim light condition (control). Samples (S) collection started at 7 PM and finished 1 hour after usual individual bed time. B) Experimental lights conditions. 3 hours in dim light, 2 hours under experimental (3000 K or 5700 K) light exposure and one additional hour in dim light. Saliva samples for melatonin assay were taken every 30 minutes. C) PLR protocol. After 5 minutes under dim light and 10 minutes in total darkness, subjects were exposed to 1-second light pulse followed by 1 minute in darkness.

Melatonin suppression tests were performed during three consecutive weeks, one day per week and the same day each week for each volunteer. In the first week, participants were instructed to follow a DLMO protocol [20, 49] at home (Fig 2A). They were asked to remain seated under dim light (<10 lux, provided by a single lamp placed as far as possible from them), every 30 minutes collecting their own saliva samples in Salivette**©** tubes (*SARSTEDT AG & Co*. *KG*, *Germany*), starting at 7 PM until one hour after their usual sleep time. During the saliva sampling they were allowed to eat and drink water after each sample but not during the previous 15 minutes to the next one. They were also allowed to read or talk, but not to use light emitting devices, including television. Participants were asked to store samples in the fridge until the following day, when samples were brought to the laboratory in ice-cooled bags and then, frozen at -20°C. The light levels to which the volunteers were exposed during the DLMO protocol were recorded by the Kronowise 3.0 (Kronohealth SL, Murcia, Spain) device, confirming the compliance of the protocol in all cases except one. This male subject was dropped off the melatonin study due to non-observance of the protocol, since he was exposed to bright light during DLMO protocol.

The second week, the participants were asked to come to the laboratory the same week day when they performed the DLMO at home, in groups of eight, and they were exposed to the first light protocol (Fig 2B). This protocol consisted of 3 hours under dim light (<10 lux), to allow the onset of melatonin secretion; 2 hours under the experimental light on (3000 K or 5700 K); and, again, one hour in dim light, to explore whether melatonin secretion resumed after light exposure. Saliva samples were collected while seated every 30 minutes, starting at 7:30 PM or 8 PM, depending on their self-reported sleep time (from the Munich Chronotype Questionnaire [50]), until 6 hours later.

The remaining light condition was assessed, again the same week day, in a third week and the order of light conditions was randomized. During the experimental nights at the laboratory, eating or drinking water were not allowed during the previous 15 minutes before each sampling point. Participants were allowed to talk, to read, to study and to play board games, but not to use smartphones or other light emitting devices. In case they needed to use the toilet, they had to wear orange sunglasses (model G 140, Wed’ze, from Decathlon, Villeneuve-d’Ascq, France) to block blue light as much as possible. None of them did this during the tested light exposure.

Saliva melatonin was quantified by radioimmunoassay (Stockgrand Ltd., University of Surrey, Guildford, UK). The intra-assay coefficients of variation (CV) for the low (mean ± SD, 5.1 pg/ml ± 0.6 pg/ml), medium (28.9 pg/ml ± 3.3 pg/ml) and high (57.4 pg/ml ± 3.6 pg/ml) pools were 11.8, 11.5 and 6.3% respectively, and limit of detection was 0.8 pg/ml ± 0.4 pg/ml. All samples collected by each volunteer were measured by duplicate in a single assay.

Then, area under the curve (AUC) for the three situations (dim light, 3000 K and 5700 K) was calculated in percentage during the light-on period, considering dim light situation as reference (0% of suppression) and determining suppression when exposed to 3000 K and 5700 K.

### Pupillary light reflex

Pupillary light reflex (PLR) was measured using commercial equipment (ViewPoint EyeTracker, Arrington Research Inc., Scottsdale, USA). It consisted of adjustable brackets, to avoid any movement during measurements, and a recording system. It is composed by an infrared camera to record in darkness, and its corresponding tracking software that allows to track participant’s pupil and to record its size. A small red LED was located behind the camera as a visual clue for the volunteer to keep their glance fixed [44, 45]. Light stimuli were applied through a cylinder (101 cm long, 20 cm diameter) and an eye piece (4 cm diameter) [51].

The protocol for light presentation is shown in Fig 2C, with all light stimuli being tested during the same session. The participant arrived at the laboratory between 9 AM and 2 PM and remained seated in dim light for 5 minutes to allow gradual pupil adaptation to darkness. Then, light was turned off for 10 minutes, starting pupil baseline recording during the last minute. The participant was asked to move and blink as little as possible. The first experimental light was presented as 1-second stimulus, after one minute of baseline recording in darkness, and recording was continued for 1 minute to assess post-illumination pupil response (PIPR). After that, the subject was allowed to rest for 3 minutes in complete darkness until the procedure was repeated for the next experimental light. The order of presentation of the two experimental light conditions, 3000 K or 5700 K, was randomized.

Blinking artefacts were removed using PupiLabWare® software. The baseline pupil diameter was quantified by averaging diameter values during the baseline recording (visually checking stability). Then, maximum constriction was also assessed with respect to basal size, as well as velocity of pupil contraction, calculated by dividing the difference of pupil diameter at baseline with the minimum diameter by time (in seconds) to reach it. We also calculated post-illumination pupil response 6 seconds after the end of the stimulus (6s-PIPR) [9, 45, 51, 52], also expressed as relative constriction with respect to basal size (0%). In addition to 6s-PIPR, post-illumination pupil response 30 seconds (30S-PIPR) after light offset [47, 53]. General standards for pupillography [54] have been followed for recording and analysis process.

### Mathematical modelling

The circadian light model proposed by Rea et al. (2010) [42], the most commonly accepted model to estimate melatonin suppression, was compared with our experimental results.

In addition to the lights experimentally tested in this work (3000 LED and 5700 LED), the model was also applied to a wide variety of commonly used indoors or outdoors light sources at different intensities, ranging from 0 to 1000 lux at intervals of 50 lux. The spectra of these sources (RGB LED, RGV LED, amber LED, fluorescent and mercury vapor) were analyzed in our laboratory by spectroradiometry (Ocean Optics Inc., Florida, USA) (Fig 1B–1F).

### Statistical analysis

Shapiro-Wilk tests were performed on the percentage of melatonin suppression in comparison with dim light condition, and on the percentage of pupil constriction, to check for data normality, and results supported the null hypothesis. Student’s t tests for related samples were applied to analyze PLR parameters and melatonin suppression, and to compare our melatonin results with those predicted by the theoretical model. To investigate sex differences, Student’s t test for independent samples were carried out with melatonin suppression and PLR parameters. To explore the relation between melatonin suppression and pupil light reflex, Pearson’s bivariate correlations were performed. Bootstrapping was performed in order to reduce the possible impact of sample size on the lack of significance. All statistical tests were carried out using IBM SPSS Statistics for Windows, Version 23.0. (IBM Corp. Armonk, NY, USA).

## Results

We carried out two different experimental approaches to test the effect on melatonin suppression and pupil light reflex of two different lighting conditions, exploring the relationship between these two circadian indexes. Moreover, we compared our experimental results with previously published theoretical models.

### Melatonin suppression

**Fig 3:**
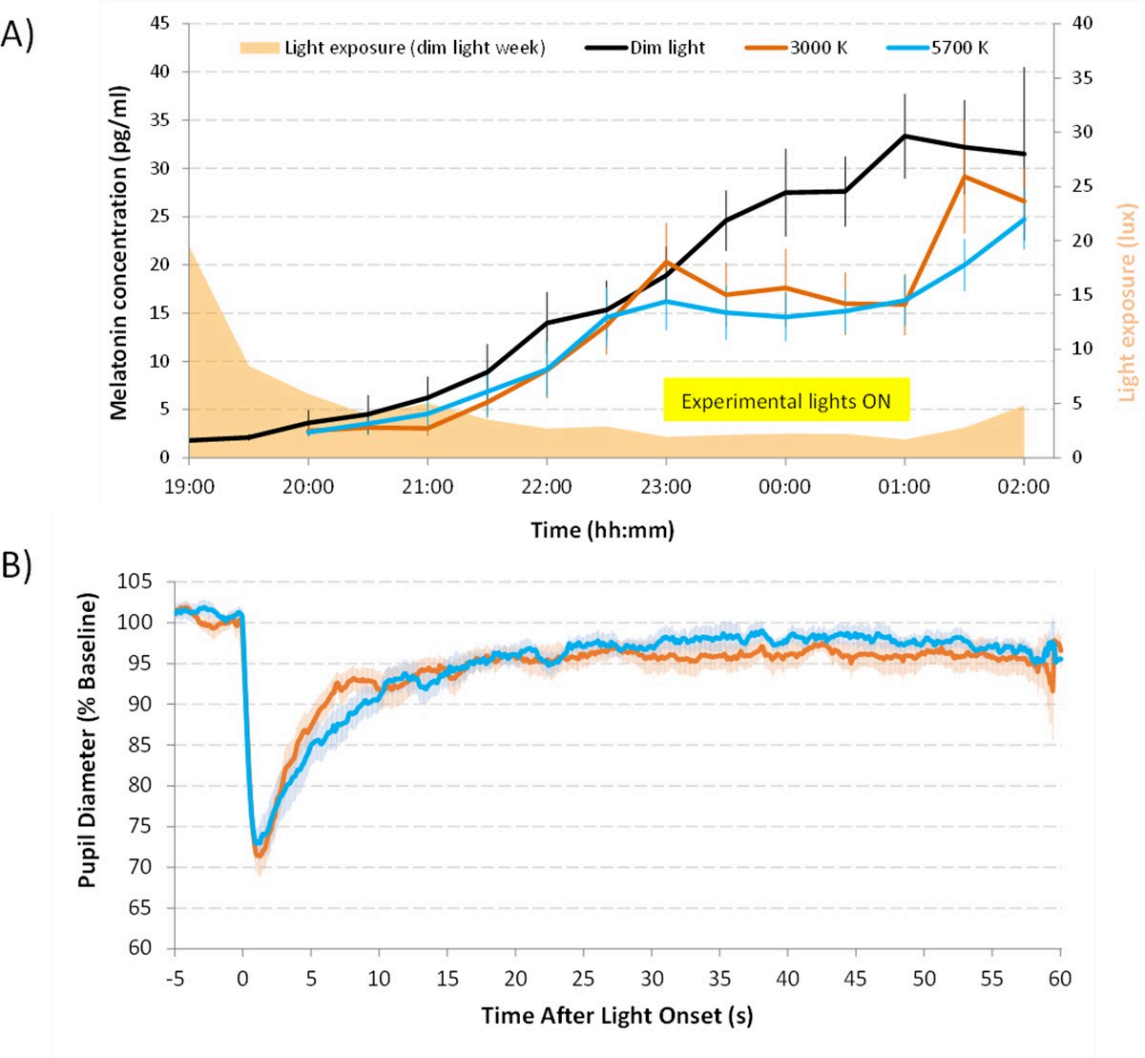
(A) Averaged salivary melatonin concentration (N = 15) under the three light conditions: Dim light (black), 5700 K (blue) and 3000 K (red). Orange area represents mean light intensity in lux, recorded during dim light condition that illustrates protocol compliance when obtaining samples at home. (B) Averaged pupillary light (PLR) reflex recordings. Pupil diameter is expressed as percentage of each recording baseline. Pale lines represent SEM. Yellow box indicates the experimental light-on period. Data are expressed as mean ± SEM.

**Fig 4:**
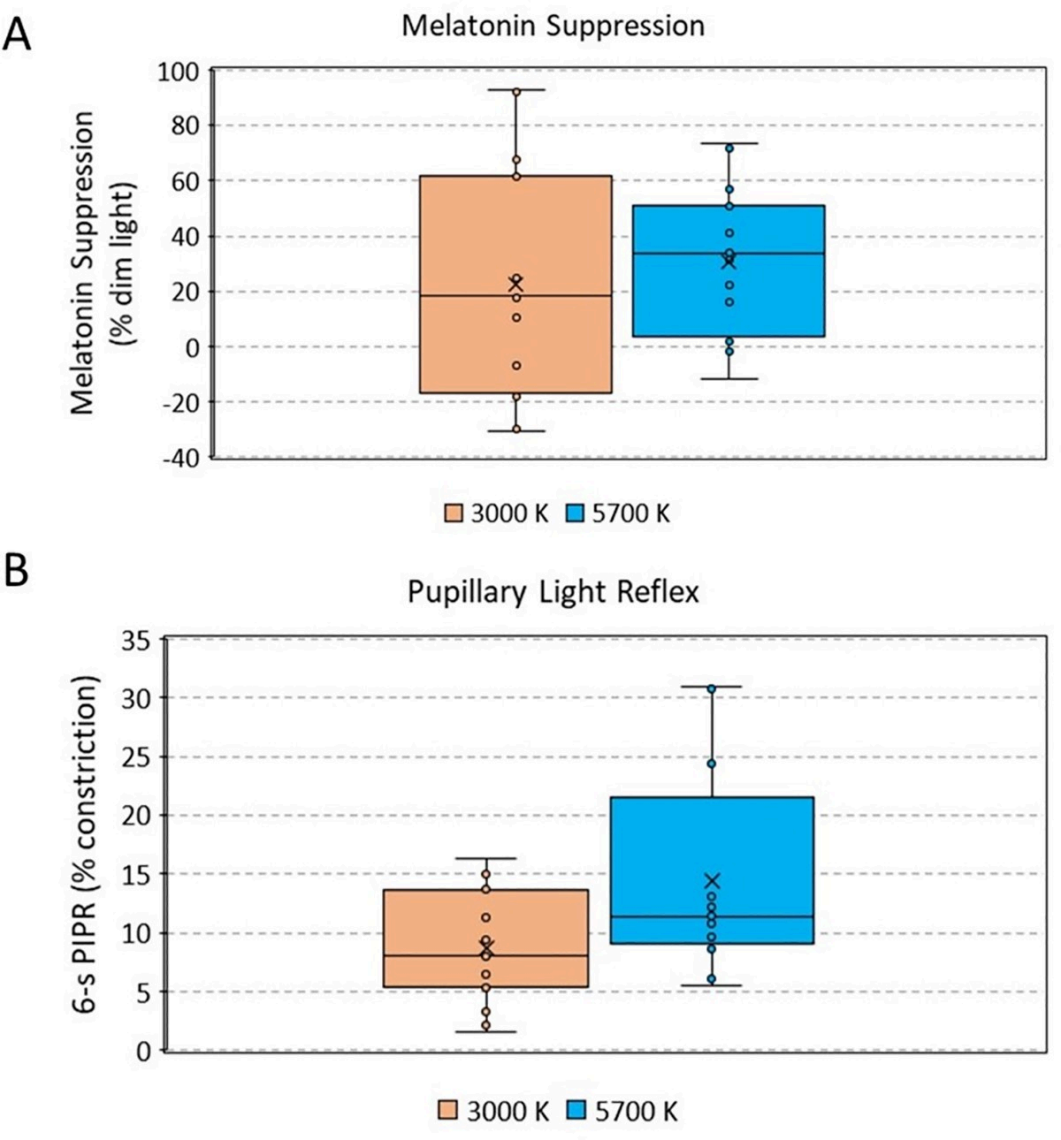
Experimental melatonin suppression in comparison with dim light (control condition, 0% of suppression, N = 15) (A) and pupil constriction 6 seconds after illumination (6s-PIPR) (N = 16) with respect to baseline (100%) under both experimental light conditions (3000 K, orange; 5700 K, blue) (B). The upper and lower whiskers indicate maximum and minimum data points, respectively. The middle line of the dataset indicates the median (second quartile), and the lower and upper half of the dataset, the first and third quartiles, respectively. Each point indicates an individual value. No significant differences were found between males and females (p = 0.756, t = 0.317, DF = 14 for 3000 K light, p = 0.730, t = 0.353, DF = 14 for 5700 K light).

Saliva melatonin was assessed in fifteen subjects (9 females, 23.8 ± 3.12 yr old, mean ± SD) under two hours of 5700 K and 3000 K lights at 569 vs 581 lux in illuminance, 184 vs 181 μW/cm^2^ in irradiance and 4.97·10^18^ vs 5.18·10^18^ photons/cm^2^/sec in photon flux, respectively, after three hours of dim light. As expected, both experimental light conditions inhibited melatonin secretion with respect to dim light condition (Figs 3A and 4A), and although suppression tended to be slightly greater under the 5700 K light (cold light) than under the 3000 K one (warm light), statistically significant differences between the two experimental light conditions were not found (Fig 4A, Student t test, p = 0.361, t = -0.944, DF = 14). As already indicated, bootstrapping was applied to data in order to palliate a possible effect of low sample size, with no changes in terms of significance.

However, the range of melatonin suppression values (compared to dim light) was wider under the warmer (3000 K) than under the cooler light (5700 K) (Fig 4A). A great intra-individual variability was observed, since 5 participants presented higher (7–31%) melatonin concentrations under warmer condition than under dim light, while only 2 of them showed higher (1–12%) melatonin concentrations under cooler (5700 K) than dim light condition (Fig 4A). We did not find any significant differences by sex (p = 0.508, t = 0.681, DF = 13 for 3000 K light, p = 0.245, t = 1.281, DF = 13 for 5700 K).

### Pupillary light reflex

In order to estimate the effects of both light conditions on ipRGCs activation, we assessed PLR (Fig 3B). Maximum constriction (minimum diameter) under 3000 K and 5700 K light stimuli was similar (31.5 ± 1.8 and 31.4 ± 1.9%, respectively, p > 0.05). However, cooler (5700 K) light yielded greatest post-stimulus contraction 6 seconds after the end of the stimulus, (6s-PIPR) than warmer (3000 K) (13.3 ± 1.9% vs 8.7 ± 1.2%, respectively, p < 0.01, t = -3.221, DF = 15) (Fig 4B).

With respect to the additional parameters, apart from 6S-PIPR, differences between the two light conditions did not reach statistical significance (p = 0.731, Student’s t = 0.351, DF = 15 for 30S-PIPR; p = 0.957, Student’s t = -0.055, DF = 15 for maximum constriction; and p = 0.251, Student’s t = -1.195, DF = 15 for velocity of constriction).

### Melatonin suppression and PLR relationship

To explore the possible relationship between PLR and melatonin suppression, correlations between them were performed. However, none of the PLR parameters analyzed in this study were found to be significantly correlated with melatonin suppression values (r = -0.209, p = 0.267 for 6S-PIPR; r = -0.170, p = 0.370 for 30S-PIPR; r = -0.115, p = 0.546 for minimum diameter; and r = -0.170, p = 0.368 for velocity of constriction) (S1 Fig).

### Experimental melatonin suppression and theoretical model

We compared our experimental results on melatonin suppression with those predicted by the most commonly used model, under the same light conditions (Table 1). Although melatonin suppression seems to be overestimated, prediction according to Rea et al.’s model [42] for warm and cold lights yielded a 3% difference between theoretical melatonin suppression under both lights, which is quite similar to the ~ 8% experimentally obtained. In fact, no significant differences between the mean experimental melatonin results and those predicted by the theoretical model according to each light condition were found (p > 0.05, t = 4.700, DF = 1 for 5700 K light and p > 0.05, t = 3.080, DF = 1 for 3000 K light).

**Table 1:** Experimental and theoretical melatonin suppression for experimentally tested light sources.

Light (CCT)	Experimental Melatonin suppression (% dim light)	Theoretical melatonin suppression. Rea et al. (2010) [42] CS %
5700 K	30.5 ± 6.7	47
3000 K	22.4 ± 10.5	44

Percentages of experimental (n = 15) and theoretical melatonin suppression under both tested light conditions (5700 K and 3000 K). Experimental data are expressed as mean ± SEM.

### Expected results for other commonly used light sources

**Fig 5:**
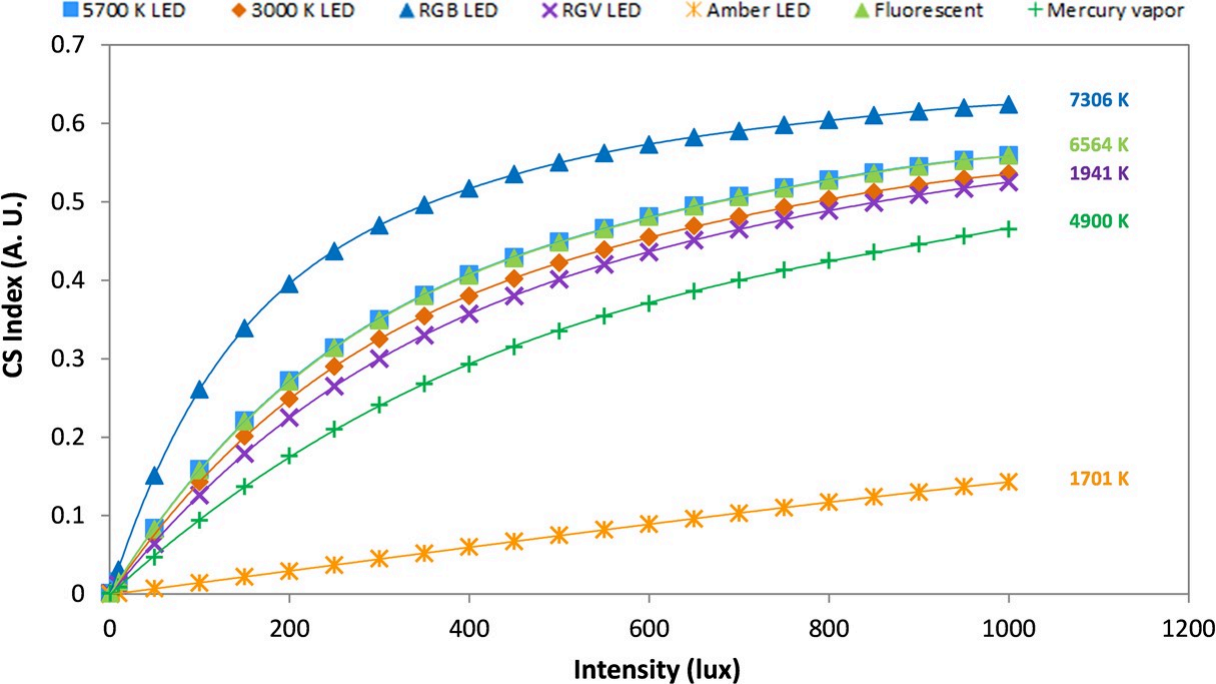
Simulations of melatonin suppression (equivalent to CS index) according to the Rea´s circadian light model [42] applied to different light sources and intensities. Light sources simulated were: the same two light conditions experimentally tested in this study (5700 K, blue square, and 3000 K LED, brown diamond, model squared Inspire 48W Gdansk, Leroy Merlin S.L., Lille, France), a red, green and blue monochromatic LED light (RGB, blue triangle, 7306 K), and a red, green and violet monochromatic LED light (RGV, purple cross, 1941 K) both of them manufactured at the University of Murcia electronic facilities, a commercial amber LED (orange cross, 1701 K, from Ignialight Sacopa S.A.U., Spain), a commercial fluorescent (green triangle, 6564 K, from ADEO, France) and mercury vapor (green cross, 4900 K, from Luxten, Romania) bulbs.

We also evaluated the theoretical melatonin suppression (estimated by Rea’s model) in a variety of light sources (for spectra, Fig 1B–1F), including those experimentally tested here, ranging from 0 to 1000 lux by 50 lux increments (Fig 5). Surprisingly, 3000 and 5700 K lights yielded very similar indexes across the intensity range considered. However, amber LED (1701 K) and RGB LED (7306 K) yielded the lowest and highest theoretical melatonin suppression, respectively.

## Discussion

The tested cooler light source produced greater sustained pupil constriction (6s-PIPR) than warmer (lower CCT), confirming greater ipRGC activation under the cooler light. However, although the higher color temperature also tended to produce greater melatonin suppression, in this case we did not detect statistically significant differences between these two light sources, which may be related to the high variability in melatonin suppression, especially under the 3000 K light source. Also, with our experimental design, no correlation was found between melatonin suppression and PLR, which may support the existence of two different ipRGCs subpopulations, each innervating the SCN or the OPN. This would also suggest that both techniques could be considered as complementary techniques in certain contexts, but not exchangeable. Also, the experimental physiological effects of the tested light sources were lower than those predicted by circadian photoreception models.

Although our results agree with some previous studies using even wider ranges of CCTs [55, 56], our suppression seemed to be lower than the ~50% found in previous studies with even lower irradiances [55, 57–59], probably due to a shorter light exposure duration. However, our results agree with those studies in terms of the high variability in melatonin suppression data. Contradictory results have been also found in other works that also explored the effect of reducing the blue part of the spectrum with no CCT change [56, 60]. Other experiments have found melatonin suppression results almost negligible for CCT < 2000 K, while increasing with CCT [61]. Our experimental design presents some limitations in terms of melatonin suppression. First, although DLMO and bed time can be related, the light exposure was scheduled based on self-reported bed time instead of on circadian phase, which could have had an impact on melatonin secretion and suppression. Secondly, the relatively high light intensities, as well as a relatively short exposure applied in these experiments could have influenced the absence of statistically significance between both tested light sources.

Regarding PLR, although the contribution of each photoreceptor type is still a matter of discussion, many studies agree that rods and cones mainly contribute to rapid and transient response [12, 47], while ipRGCs mainly regulate the sustained pupilloconstriction after light offset (post-illumination pupil response, PIPR) [9, 11, 52]. Namely, 6s-PIPR (PIPR 6 seconds after light offset) has been considered a good marker for ipRGC activation [9]. Indeed, our 6s-PIPR results are in agreement with previous PLR studies using polychromatic, as well as single or combined monochromatic sources [45, 51], with greater 6s-PIPR under cooler (higher blue content) than warmer (lower blue content) lights.

However, pupillometry, although rapid and relatively easier and cheaper technique than melatonin assessment, could not replace the information obtained with this latter. The fact that we did not find any significant correlation between both processes could be related with the existence of different subpopulations of Type 1 ipRGCs. Indeed, it has been described that both the SCN and OPN are innervated by M1 ipRGCs in mice (and thus, both PLR and melatonin suppression are mediated by melanopsin [62]). However, it has been reported that among M1 subtype, Brn3b+ subpopulation would innervate the OPN, while Brn3b- subpopulation would project to the SCN. In this sense, our group has previously reported a negative correlation between circadian robustness and PLR parameters, with reduced pupil constriction for subjects with more robust rhythms [44]. Although in the current work no correlation was found (either positive or negative), we consider that the different distribution of Brn3b+ and Brn3b- subpopulations could be behind this absence of quantitative relationship. It should also be noted that melatonin suppression also entails other processes that involve, not only transmission of the light stimulus, but also a complex physiological response at the SCN and the pineal gland level. In this sense, Rahman et al. (2018) [63] established that melatonin suppression and phase resetting are sometimes correlated, but generated by distinct processes down-stream of retinal signal transduction.

Apart from those physiological differences between both processes, our study presents some limitations that could have influenced the absence of correlation between PLR and melatonin suppression. First, PLR was assessed during the morning, while melatonin suppression tests were evidently performed during the evening-night. These tests were not scheduled based on individuals’ circadian phase, which may have an impact on our results, since PLR is time-of-day dependent. In this sense, previous studies conclude that the intrinsic melanopsin system becomes less sensitive to light in the second half of the night, after the peak of melatonin secretion and closer to wake time, depending on light wavelength [64]. On the other hand, the participants were asked to maintain regular sleep habits, although the compliance could not be verified, so the possible effect of previous irregular sleep history might be affecting both melatonin suppression and PLR results [65, 66].

Another limitation of our study that could have influenced this lack of correlation is the relatively high light irradiance used. Also, although the two CCT tested were selected in terms of their commercial use, a wider range of CCT and intensities would be desirable. Also, the fact that in PLR, testing subjects were adapted to darkness, while in melatonin suppression tests they were adapted to dim light, may have contributed to the lack of correlation, although these protocols have been previously used in literature. In any case, although we cannot rule out the possibility that these limitations contributed to the absence of correlation, considering previous results, we suggest that melatonin suppression and pupil light reflex should be complementary techniques, since they do not provide the same information. This is in consonance with other authors that suggest that it is unlikely that a single method could adequately describe the spectral sensitivity of all melanopsin RGC-driven responses under all conditions [62].

Considering that Rea et al.’s model fitted our experimental results in an acceptable manner and in order to facilitate extrapolation from our limited experimental data to a wider range of light sources and intensities, theoretical simulations on melatonin suppression were performed. Apart from the expected differences among light sources with very different spectra (e.g. amber and RGB LEDs), we found extremely similar predictions for 3000 and 5700 K light sources. These differences became even smaller at intensities below 200 lux and above 500 lux, suggesting that light intensity should gain more importance than CCT when designing nocturnal lighting systems. Thus, if we progressively reduce the intensity of a cool light (e.g. 5700 K) towards the night, we could decrease its deleterious effects on melatonin secretion in a greater manner than just by replacing it with a warmer light (e.g. 3000 K). This could be essential when considering indoors nocturnal light, that can easily reach around 200 lux [67], thus eliciting suppressions from 3% for amber LED to 40% for RGB LED.

When designing nocturnal light environments, other objective light exposure characteristics (e.g. intensity, spectrum, direction, duration and time) should be considered. In this sense, previous studies have reported that longer exposures and higher intensities produce greater melatonin suppression [56]. However, other works describe that non-visual responses could be provoked by light exposures as short as 1–5 minutes of duration and around 90 melanopic lux [59, 68]. This fact, together with the high interindividual variability in the circadian responses [69], make necessary further investigations in this area.

Comfort is a subjective factor that should also be considered in lighting field. In this sense, the comfort produced by a certain CCT has been found to be dependent on light intensity [38, 70], with higher intensities being preferred for cooler lights, while warm lights are perceived as more comfortable when applied at lower intensities. Therefore, the combination of both characteristics may play a crucial role [71]. A more recent study revisiting Kruithof’s rules for LEDs reported, however, a relative increase of brightness with CCT [70], together with greater comfort under low CCT at any illuminance (150, 300 or 600 lux). Also other studies suggest that the variation in CCT between 2500 and 6500 K does not affect pleasing conditions [39]. All this could suggest that reducing the actual light intensity in cooler lights may reduce its deleterious physiological effects with almost no effect on the subjective perception of the light.

Our study highlights the importance of considering, not only CCT, but also intensity when designing lighting systems. In this sense, our “warm” light produced similar melatonin suppression as the “cool” light in our participants. Also, our experimental results point out to pupillometry as a complementary technique that can contribute to the understanding of non-visual light effects. With the limitations stated, our results indicate the possibility of two different subpopulations of ipRGCs innervating the OPN and the SCN, which would open the way for further research in terms of ipRGCs projections. According to our results, it would be recommended to use warm colours at the lowest possible intensities for nocturnal lighting, since a 3000K also produces a reduction in melatonin secretion. Diurnal artificial illumination, on the contrary, should rely on higher intensity and cooler lights (high CCT). More studies with different experimental designs and covering a wider range of CCTs, irradiances and exposure durations should be performed to confirm these results.

## Supporting information

S1 Fig Scatter plots for PLR parameters (6s-PIPR, 30s-PIPR, maximum constriction and velocity of constriction) and melatonin suppression under 3000 K (orange) and 5700 K (blue). (TIF)

S1 Data (XLSX)
