# Supplementary figures and images for: Correlated color temperature and light intensity: Complementary features in non-visual light field
Source: PLoS One. 2021 Jul 12;16(7):e0254171. doi: 10.1371/journal.pone.0254171 (PMC8274909; doi:10.1371/journal.pone.0254171)

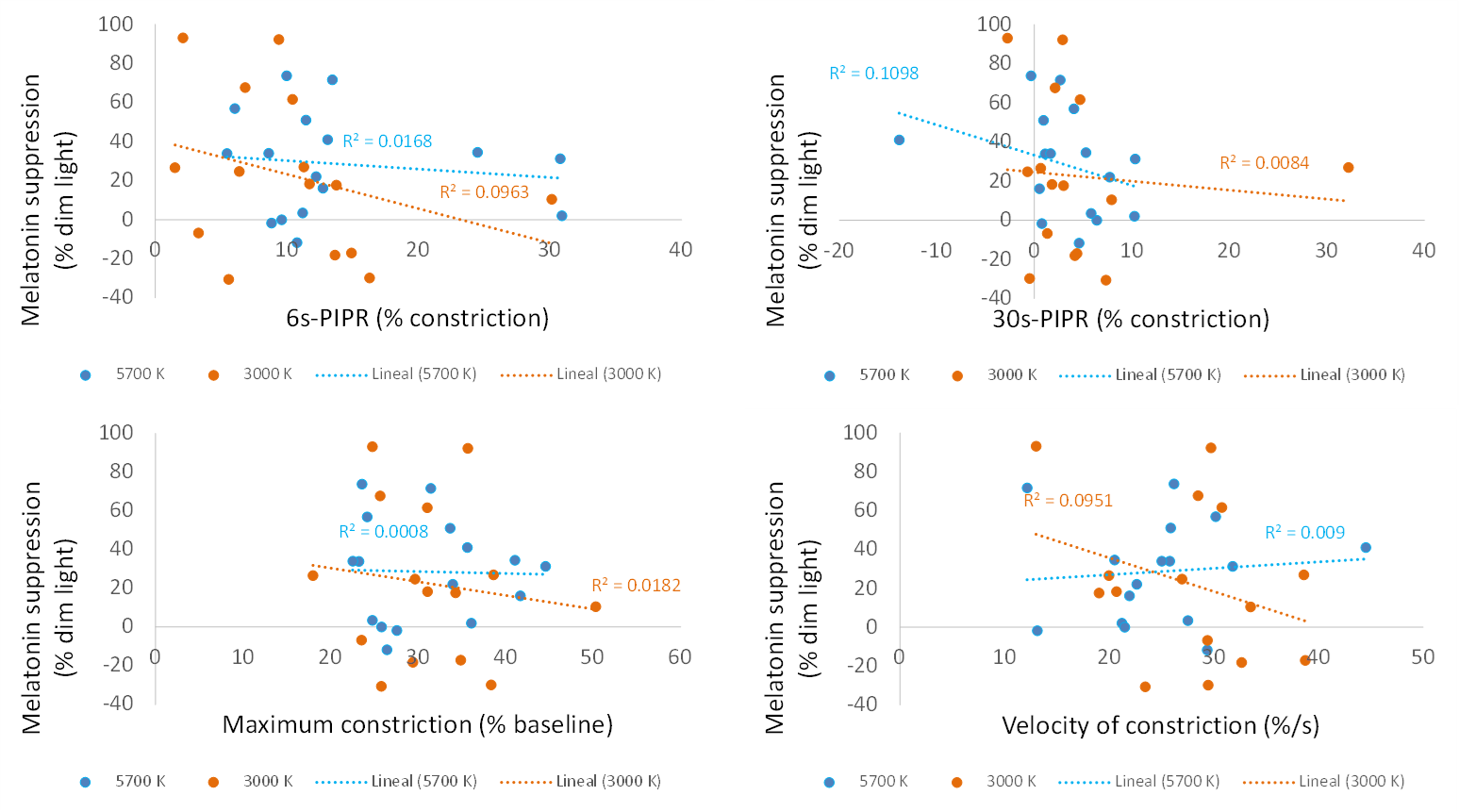

Supplement: S1 Fig — (TIF) [file pone.0254171.s001.tif]
